# A Composite Sketch of Fast-Spiking Parvalbumin-Positive Neurons

**DOI:** 10.1093/texcom/tgaa026

**Published:** 2020-06-19

**Authors:** Odile Bartholome, Orianne de la Brassinne Bonardeaux, Virginie Neirinckx, Bernard Rogister

**Affiliations:** 1 GIGA-Neurosciences, University of Liege, 4000 Liège, Belgium; 2 Neurology Department, CHU, Academic Hospital, University of Liege, 4000 Liège, Belgium

**Keywords:** basket cell, epilepsy, fast-spiking cell, parvalbumin neuron

## Abstract

Parvalbumin-positive neurons are inhibitory neurons that release GABA and are mostly represented by fast-spiking basket or chandelier cells. They constitute a minor neuronal population, yet their peculiar profiles allow them to react quickly to any event in the brain under normal or pathological conditions. In this review, we will summarize the current knowledge about the fundamentals of fast-spiking parvalbumin-positive neurons, focusing on their morphology and specific channel/protein content. Next, we will explore their development, maturation, and migration in the brain. Finally, we will unravel their potential contribution to the physiopathology of epilepsy.

## Introduction

The brain is constituted of several cell types, interacting together in a fine-tuned network to allow individuals to perform complex tasks. Novel cellular and molecular actors in this neuronal network are continuously brought to light, increasing the complexity of the yarn we are trying to unravel. The human brain contains approximately one hundred billion neurons and roughly as many glial cells (von Bartheld et al. 2016). In mice, the balance is more tilted towards neurons (Herculano-Houzel et al. 2006). Although it is challenging to assess the proportion of each cell population of the brain, it is considered that 80% of neurons in the mouse brain are excitatory and 15% of them are inhibitory (Keller et al. 2018). The remaining 5% fall into several specific neuronal populations, whose description is beyond the scope of this review.

The considerable heterogeneity of the brain has been known for over a century, and inhibitory neurons have been particularly targeted by many classification attempts based on markers, electrophysiological profile(s), connectivity, morphology or other characteristics. These various endeavors indicate in fact that their identification and classification remain unsatisfactory. None of the aforementioned features is sufficient to distinguish an interneuron *per se*, and inhibitory neurons are more intricate than excitatory neurons. Thanks to single-cell RNA sequencing, efforts to sort inhibitory neurons by gene expression recently resulted in even more molecular subclasses than previously suggested (Zeisel et al. 2015; Tasic et al. 2016; Tasic et al. 2018). Interestingly, these subclasses seem universal as they can be found almost in all areas of the brain, whereas many glutamatergic neuronal groups differ from one brain region to another (Zeisel et al. 2015; Tasic et al. 2018). Inhibitory neurons are usually divided into 3 subpopulations, based on molecular markers: they are either positive for parvalbumin (PV—a calcium-binding protein), somatostatin (SOM—a neuropeptide), or 5HT_3_ (a serotonin receptor). Besides, inhibitory interneurons can be distinguished by the expression of other calcium-binding proteins such as calbindin (CB) or calretinin (CR), other neuropeptides as cholecystokinin (CCK), vasoactive intestinal peptide (VIP), or neuropeptide Y (NPY) (Defelipe et al. 2013). In this review, we will focus on the parvalbumin-positive neurons (PV+ neurons), and more specifically on PV+ neurons from the cortex and the hippocampus.

## Discussion

### General Features of Fast-Spiking PV+ GABAergic Interneurons

PV+ neurons are one of the most abundant subtypes of GABAergic interneurons, accounting for 30%–40% of them (Tremblay et al. 2016). These neurons are usually characterized by fast-spiking profile and can be found almost everywhere in the brain. The term “*fast-spiking*” (FS) refers to the firing pattern of those cells. It is associated with short-action potentials and the capability to sustain a high firing frequency. Having a closer look at the available tools for studying PV+ neurons, one could notice that those cells are built for speed in almost every aspect of the transmission.

Based on their axonal arborization, PV+ interneurons of the cortex are morphologically described either as “basket cell” (BC) or “chandelier cell” (ChC, also named axo-axonic cell) (Katsumaru et al. 1988a; DeFelipe et al. 1989; Hendry et al. 1989). While almost all fast-spiking BCs are PV+, things are more complicated for ChCs. With their typical morphology and low number, ChCs rarely required any specific labeling, so the observation that only a fraction of ChCs is PV+ was made quite recently (Taniguchi et al. 2013). In the cortex, PV + -ChCs seem to express vasoactive intestinal peptide receptor 2 (Vipr2) as a secondary marker and a recent study identified the protein Fgf13 as expressed by virtually all ChCs (Tasic et al. 2018; Favuzzi et al. 2019). Vipr2 is a G protein-coupled receptor for the corresponding neuropeptide, while Fgf13 is a multifunctional protein implicated in microtubule stabilization for instance.

**Figure 1:**
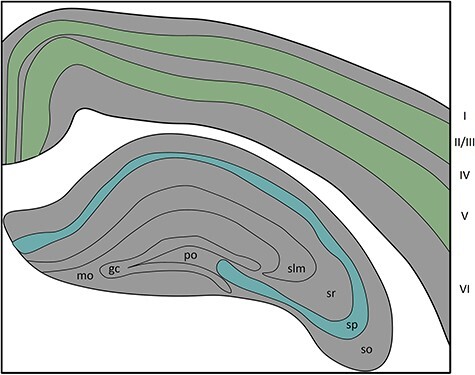
Schematic view of layers most commonly occupied by PV+ neurons in the cortex (green) and the hippocampus (blue). I–VI: layers of the cortex, so: stratum oriens, sp: stratum pyramidale, sr: stratum radiatum, slm: stratum lacunosum-moleculare, po: polymorphic layer, gc: granule cell layer, mo: molecular layer.

BCs and ChCs are also found in the hippocampus, along with 2 other PV+ populations: *oriens-lacunosum moleculare* cells (O-LM) and bistratified cells (BiC) (Halasy et al. 2002). BCs, ChCs, and BiCs can be observed mainly in *stratum pyramidale* (Fig. 1), while O-LMs are found in the *stratum oriens* of the hippocampus (Yamada and Jinno 2017). However, as O-LMs and BiCs also express other markers, we will not go into further details about them (Somogyi and Klausberger 2005; Jinno and Kosaka 2006).

### Mode of Action of PV+ Neurons

#### Recruitment Mechanisms: The Dendritic Tree

**Figure 2:**
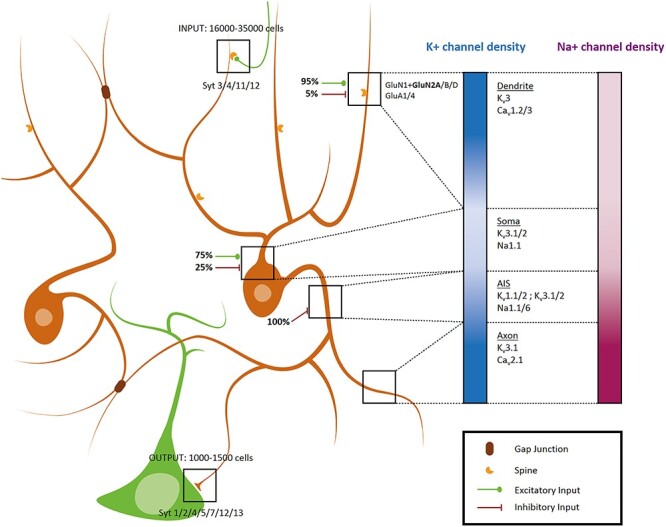
Schematic view of mature PV+ neurons (orange) and their major known features. One pyramidal neuron is in green. Blue and purple gradients symbolizing the concentration of, respectively, K+ and Na+ channels in the corresponding compartment of the neuron defined by black squares and dotted lines.

PV+ cells display one of the most extensive dendritic trees among interneurons, as well as the largest number of received inputs, most of them being excitatory (Fig. 2). In the hippocampus, PV+ cells receive between 16 000 and 35 000 contacts mainly originating from pyramidal cells (PC) or granular cells (GC), only around 10% are inhibitory (Gulyás et al. 1999; Tukker et al. 2013). The dendritic tree of cortical PV+ neurons displays a low density of spines with excitatory synapses (Sancho and Bloodgood 2018), the majority of inputs being received on dendritic shafts (i.e., axo-dendritic contact). A small proportion of those axo-dendritic inputs come from SOM+ or other PV+ interneurons. Inhibitory axo-somatic inputs are mainly coming from VIP+ interneurons, whereas axo-axonic contacts are probably arising from ChCs (Hioki et al. 2013; Tukker et al. 2013). One recent study has reported that PV+ neurons with local high spine density, associated with few perineuronal nets (PNN, see Maturation), could be found in the dentate gyrus. (Foggetti et al. 2019).

In terms of glutamate receptors, it has been shown that the post-synaptic compartment of PV+ neurons presents different types of NMDA and AMPA receptors (NMDAR and AMPAR) to suit their specific electrophysiological profile. NMDARs differ from each other in their diverse subunits and therefore exhibit different decay kinetics (Tovar and Westbrook 2017). Adult cortical and hippocampal PV+ neurons mainly rely on the GluN1 and GluN2A NMDAR subunits, the duo with the fastest decay kinetics, but the association of GluN1-GluN2B and GluN1-GluN2D (in the hippocampus only) can also be found (Monyer et al. 1994; Geiger et al. 1997; von Engelhardt et al. 2015; Mierau et al. 2016). NMDARs are involved in calcium influx when Mg^2+^ blockade is alleviated and may be linked to PV and GAD67 expression in the cortex (Kinney 2006). Interestingly, NMDARs tend to be concentrated in spiny synapses, in contrast to AMPARs, which are uniformly distributed along the dendrites of cortical PV+ neurons (Sancho and Bloodgood 2018).

AMPARs are also made of different subunits, conferring specific gating and kinetics to the complex (Lee 2012). In cortical and hippocampal PV+ neurons, it has been shown that the majority of AMPARs lacks the GluA2 subunit, making it permeable to Ca^2+^ (Jonas et al. 1994; Geiger et al. 1995; Talos et al. 2006). These calcium-permeable AMPARs are largely responsible for Ca^2+^ influx into the postsynaptic compartment. They also ensure NMDAR activation through the clearance of Mg^2+^ (Goldberg et al. 2003b; Camiré and Topolnik 2014). AMPARs allow PV+ neurons to quickly generate excitatory post-synaptic potential (EPSP) during a short period following excitatory inputs. Because one EPSP is rarely enough to induce an AP generation, this system allows PV+ neurons to fire only if they received several synchronous EPSPs. Indeed, AMPARs’ conductance, rapid rise, and decay parameters associated with strong desensitization and small recovery time make them particularly suitable to the task (Geiger et al. 1997; Traynelis et al. 2010).

Aside from these glutamate receptors, EPSPs are also shaped by ions channels in PV+ neurons. At least 2 L-type Ca^2+^ voltage-gated channels can be found in hippocampal PV+ neurons, which also influence their firing properties (Jiang and Swann 2005; Xu et al. 2006). Besides, voltage-gated K^+^ channels type 3 (Kv3) are highly concentrated in cortical and hippocampal PV+ dendrites (Rudy and McBain 2001). Kv3 channels contribute to the propagation and the quick decay time of EPSP, ensuring a tight summation window for generating action potential (AP) (Fricker and Miles 2000; Hu et al. 2010). Besides, the concentration of Na^+^ channel is relatively low, specifically at the apical dendrite. This particular cocktail of ion channels explains why cortical and hippocampal BCs are unable to produce dendritic spikes. Different teams showed that short high-intensity somatic currents fail to trigger an AP in the dendrite of FS neurons, contrary to the situation observed in PCs. Upon long somatic current pulse, an AP can be observed in the basal dendrite but fails to ascend further (Goldberg et al. 2003a; Hu et al. 2010). The ascension of APs is also called “backpropagation” and probably participates to synaptic plasticity, which we will approach in the section The long-term Plasticity in PV+ Neurons. Finally, cortical and hippocampal dendrites of PV+ neurons are connected by gap junctions, further contributing to the high synchronic power of this neuronal network (Katsumaru et al. 1988b; Galarreta and Hestrin 1999).

#### Action Potential Generation: The Axonal Compartment

The signature of PV+ interneurons lies in their axonal thickness and ramifications. In cortical BCs, the axon usually originates from the apical part of the soma and extends mainly around it, targeting proximal dendrites and soma of postsynaptic cells. In cortical ChCs, the axon arises from the basal part of the soma and has a typical candlesticks-like shape, connecting to the axon of their target (Markram et al. 2004; Jiang et al. 2015). The location of synapses that connect PV+ interneurons with their targets improves their efficiency: the closer it is to the AP generation site, the stronger the synapse is (Kubota et al. 2015). Recent studies have demonstrated that most cortical BC neurons present patches of myelin sheets on their axon, both in mice and humans. Authors suggest an effect on the energy needed for the AP propagation (rather than the classical role in AP velocity), but pieces of supporting evidence are scarce (Micheva et al. 2016; Micheva et al. 2018).

All PV+ neurons initiate the AP in a structure particularly close to the soma, called the Axon Initial Segment (AIS) and characterized by a specific cocktail of ions channels that are in high densities (Hu et al. 2010; Hu and Jonas 2014; Höfflin et al. 2017). The AIS of PV+ neurons contains classical voltage-gated channels (Kv1.1, 1.2 and Nav1.6), along with some other channels (Lorincz and Nusser 2008). Indeed, the voltage-gated sodium channel Nav1.1, enriched in the AIS of all FS cells, contributes to AP generation by sustaining high-frequency firing, while Kv3.2 channels complete the AP during repolarization at least in the cortical PV+ neurons (Lau et al. 2000; Ogiwara et al. 2007). A comparison between CCK+ and PV+ neurons revealed that they use different types of channels to trigger Ca^2+^ entry into the pre-synapse. Cortical and hippocampal BCs mainly rely on Cav2.1 (also called P/Q-type) channels to release neurotransmitter rapidly following APs, in a synchronous manner. Neurons in which fusion events can occur several seconds after APs mediate Ca^2+^ entry partly or entirely using Cav2.2 (N-type) channels (Hefft and Jonas 2005; Zaitsev et al. 2007; Rossignol et al. 2013).

Similar to dendrites, axons of cortical and hippocampal PV+ neurons are connected by gap junctions, allowing them to propagate newly generated AP (Kosaka and Hama 1985; Tamás et al. 2000). Interestingly, a recent study notably reports that simultaneous or sequential excitation of several connected cerebellar BCs increased the probability of AP generation and reduced their latency (Alcami 2018).

#### Signal Transmission: Pre-Synapses and Synapses

The synapses of PV+ neurons also contribute to their ability to fire very rapidly following an input. All hippocampal interneurons apparently display a similar presynaptic terminal density (i.e., 21–28 synapses/100 μm), which means that 1 BC innervates around 1500 cells (with an average of 6 contacts per target) (Gulyás et al. 1993; Buhl et al. 1994; Sik et al. 1995). Their synapses also present a low failure rate and a rapid release of GABA following to AP arrival, again stressing out the need for synchronous communication (Kraushaar et al. 2000). Hippocampal BCs organize their synaptic machinery into small boutons, bearing a small number of active zones, considered as “nanodomains.” As a result of their spatial promiscuity, 2 or 3 Ca^2+^ channels are sufficient to induce the vesicle release via Ca^2+^ sensor protein-dependent exocytosis (Bucurenciu et al. 2008; Bucurenciu et al. 2010). These well-organized nanodomains also facilitate Ca^2+^ clearance following neurotransmitter release and save energy, as less ion exocytosis is needed to return to the resting condition.

Besides, the parvalbumin protein has a buffering ability and helps to rapidly decrease the Ca^2+^ concentration. PV is a calcium-binding protein with 3 EF-hand domain also found in muscle cells (Bottoms et al. 2004). Even if the dissociation constant of PV and Ca^2+^ is very low, this protein presents rather slow binding kinetics. Indeed, the binding capacity of PV to Mg^2+^ at physiological concentration makes it the favored partner, impeding or slowing the reaction with Ca^2+^ (Schwaller 2009). As a result, PV co-exists in 3 states: free of ions, bound to Mg^2+^ or Ca^2+^. It turns out that this three-state organization could contribute to the buffering capacity of PV. Indeed, computational studies performed on cerebellar BCs revealed that free PV is replenished only from the Mg^2+^-bound fraction, thus ensuring a constant buffer capacity during stimulation (Eggermann and Jonas 2012). The high mobility of this protein combined with the organization in nanodomains could, therefore, contribute to easier maintenance of buffering capacity, as even small changes in the absolute number of ions could impact their total concentrations (Schwaller 2009; Eggermann et al. 2012). PV could also participate in the depressing profile of BCs synapses, as suggested by the facilitation phenotype of cerebellar and hippocampal BCs in PV-deficient mice (Vreugdenhil et al. 2006; Eggermann and Jonas 2012). A depressing synapse, in contrast with a facilitated one, presents a reduced amplitude of post-synaptic current following a train of APs. Neurons with a high probability of release usually harbor depressing synapses, as their efficient machinery rapidly clear transient Ca^2+^ and/or they will use the majority of their readily releasable pool (RRP) of vesicles faster than its replenishment potential (Jackman and Regehr 2017).

#### The Synaptotagmins in PV+ Neurons

Synaptotagmins (Syt) also take part in the peculiar profile of PV+ neurons. This family of proteins includes 17 members, divided into 3 groups according to their ability to bind 0, 5, or 10 Ca^2+^ ions. Syt also differ by their binding kinetics. A majority of them are also able to bind SNARE proteins, a complex responsible for synaptic vesicles docking to the plasma membrane of neurons (Chen and Jonas 2017; Wu et al. 2019). Hippocampal PV+ neurons express 9 paralogs of the synaptotagmin family (i.e., 1–5, 7, and 11–13), although their genuine role in inhibitory neurons is unclear (Kerr et al. 2008). The fast-release sensors Syt1 and Syt2 are found in cortical and cerebellar PV+ neurons (as well as in a fraction of hippocampal BC) and ensure a fast and synchronous neurotransmitter release, contributing to the depressing profile of PV+ neurons’ synapses (Sommeijer and Levelt 2012; Bouhours et al. 2017; Bornschein and Schmidt 2019). Contrary to Syt1 and Syt2, Syt7 is not located in the vesicle membrane but at the plasma membrane of cerebellar and hippocampal PV+ neurons and is implicated in an asynchronous release, facilitation, and managing of the RRP (Jackman et al. 2016; Li et al. 2017). Even though asynchronous release and facilitation are not typical characteristics for PV+ neurons, Syt7 ensures sustainable neurotransmission by spreading vesicle release over time following one or several APs, thus prolonging inhibition (Chen et al. 2017). Besides, Syt1 and Syt7 may contribute to vesicle recycling through clathrin-mediated endocytosis (CME) and a slower calcium-independent mechanism, respectively, (Haucke et al. 2000; Li et al. 2017).

Syt11 could play a balancing role by inhibiting the CME in hippocampal and ganglionic neurons, thereby maintaining a reasonable number of active endocytosis sites in a calcium-independent manner (Wang et al. 2016; Wang et al. 2018). Other teams reported the presence of Syt11 in dendritic endosome-like structures, linking the protein to long-term potentiation (LTP, see below) rather than vesicle recycling. Indeed, Syt11 knock-out (KO) neurons display a normal secretion of neurotransmitters and peptides (Dean et al. 2012; Shimojo et al. 2019). The role of Syt4 seems to be subtler, as several experiments on neurons and neuroendocrine cells showed that Syt4 can impair vesicle fusion by unproductively competing for SNARE binding with other Syt. In contrast, it enhances exocytosis under high Ca^2+^ concentration (Wang et al. 2001; Wang et al. 2003; Bhalla et al. 2008; Zhang et al. 2009; Huang et al. 2018). These concentration-dependent opposite effects of Syt4 is surprising given its inability to bind ions (Dai et al. 2004) but could be associated with its interaction with other calcium-dependent Syt members. The absence of effects following Syt4 overexpression in hippocampal neurons may argue against its putative fusion-impairing role or highlight a failsafe mechanism preventing the system from over-inhibiting fusion (Ting et al. 2006). Another paper has shown that the absence of Syt4 in presynaptic terminals increases the spontaneous release of vesicles via BDNF, thus confirming the inhibitory effect of Syt4 on vesicle fusion (Dean et al. 2009). The same team has also reported a link between Syt4 and LTP, as observed for Syt11.

Syt12 also seems able to compete with Syt1 for SNARE binding, potentially acting as another inhibitory protein (to a lesser extent compared to Syt4, likely not biologically relevant) (Bhalla et al. 2008). By contrast, it was reported that Syt12 could support spontaneous release when phosphorylated after binding to Syt1 in an SNARE-independent manner (Maximov et al. 2007). Finally, Syt3 has recently been linked to AMPAR internalization following NMDA or AMPA activation in hippocampal neurons primary cultures (Dean et al. 2012; Awasthi et al. 2019). Removing Syt3 in mice leads to a lack of forgetting ability, but since its activity is mainly mediated by GluA2 binding (a subunit of AMPA receptor weakly expressed in BC), its effect in PV+ neurons could be limited.

Most of the information about Syt5 functions is inferred from its activity in secretory cells. Syt5 has been found in peptide-containing, dense-core vesicles from the adrenal medulla or pancreatic-derived cell lines. Syt5 could act as a positive modulator of calcium-dependent exocytosis (Saegusa et al. 2002; Iezzi 2004). To date, no genuine evidence for a defined function of Syt13 in the brain has been generated. However, the glucose-induced secretion of insulin is significantly reduced in pancreatic cells with lower expression of Syt13 (Andersson et al. 2012). Together, all these studies show the wide variety of molecular tools that PV+ neurons can use to fine tune their activity and ensure fast and efficient responses.

#### The Long-Term Plasticity in PV+ Neurons

Once generated in the AIS, the AP can backpropagate towards the proximal dendrite and usually fails to reach to the distal dendrites, probably due to the lack of Na channels. In cortical BCs, this phenomenon has minimal impact on Ca^2+^ accumulation and is regulated by A-type K^+^ channel (Goldberg et al. 2003b; Cho et al. 2010). Nonetheless, recent findings indicate that sharp wave oscillations and nicotinic cholinergic receptors (nAChR) could carry the AP further into the distal dendrite and sustain high Ca^2+^ signal in hippocampal BCs (Chiovini et al. 2010). Moreover, type I metabotropic glutamate receptor (mGluR) were reported on PV+ neuron membranes and could also contribute to Ca^2+^ accumulation in the post-synaptic compartment (Muly et al. 2003; Sun et al. 2009; van Hooft et al. 2018). Unlike axons, Ca^2+^ concentration in dendrites of hippocampal BC is mainly managed by a fixed rather than a mobile buffer. A fixed buffer should induce a slow Ca^2+^ release close to the source, elongating the decay time of transient Ca^2+^. This could provide an efficient summation of Ca^2+^ influx between 2 events and explain why successive inputs are able to accumulate Ca^2+^ in dendrite and support AP backpropagation (Aponte et al. 2008).

Once accumulated in the post-synaptic compartment, the calcium contributes to the synaptic plasticity of the neuron. The phenomenon has been mainly studied in the hippocampus, and it was shown that yet again PV+ neurons have several tools available to modulate their activity. *In vivo* experiments showed that theta burst stimulation (TBS) in rat hippocampi can either induce long-term potentiation (LTP), facilitating the next AP generation or long-term depression (LTD), inhibiting the next burst of AP generation (Lau et al. 2017). Unlike PCs, interneurons of the CA1 showed a special Hebbian LTP that is independent of NMDAR but involve group I mGluR and AMPAR (Perez et al. 2001). Further *ex vivo* experiments on mice hippocampal slices showed that subthreshold TBS (thus triggering no AP) can induce anti-Hebbian LTP through CP-AMPAR-driven Ca^2+^ accumulation (Lamsa et al. 2007; Camiré and Topolnik 2014). On the contrary, suprathreshold TBS (thus producing a signal) creates LTD, weakening the next input. In this case, the transient Ca^2+^ increase is higher and relies on internal storage in addition to CP-AMPAR contribution (Camiré and Topolnik 2014). Moreover, group I mGluR and cannabinoid receptors have also been implicated in LTD of hippocampal FS neurons (Péterfi et al. 2012). A computational study revealed that internal stores, clearance mechanisms and the specific morphology of the dendrite have a major impact on the calcium summation system of the neuron (Camiré et al. 2018).

### Development of PV+ Neurons

#### Migration

**Figure 3:**
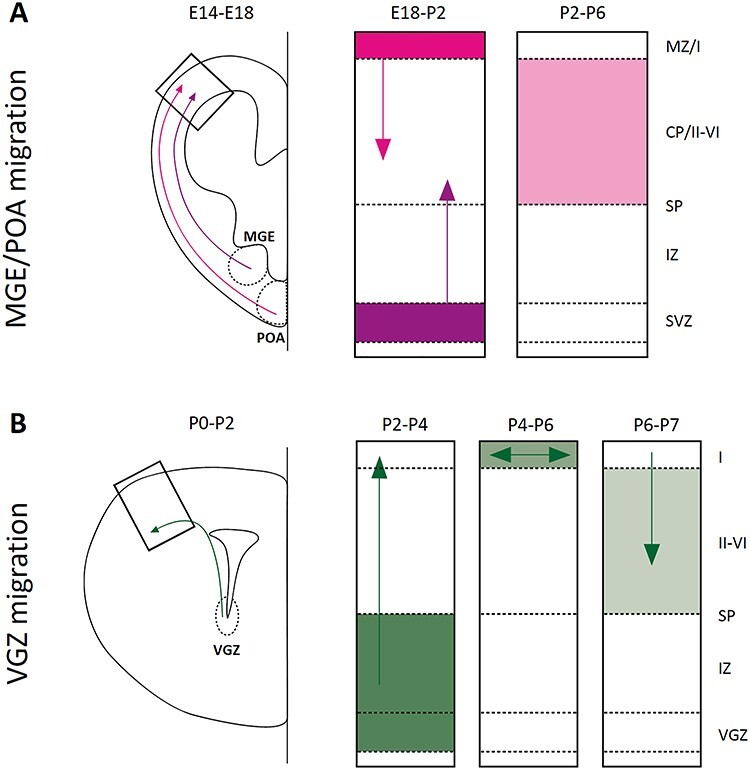
Schematic view of migration routes used by PV neurons. The first step of each route shows the migration path while the right part depicts the invasion of the cortical plate. *A*: paths from the MGE (in purple) and the POA (in pink). *B*: the path from the VGZ (in green). MZ: marginal zone, CP: cortical plate, SP: subplate, IZ: intermediate zone, SVZ: sub-ventricular zone, I–VI: layers of the cortex. Colored areas represent the population of migrating neurons and arrows represent the movement the population is taking.

During mouse development (Fig. 3), the majority of PV+ interneurons populating the cortex and the hippocampus comes from the rostral part of the medial ganglionic eminence (MGE), with weakened Wnt signaling (McKenzie et al. 2019). MGE is present from mouse embryonic day 9 (E9) to E16 and generates interneurons from E13.5, which migrate through the marginal or subventricular zone to reach their final destination (Wichterle et al. 2001; Xu 2004). A smaller proportion of fast-spiking BCs arises at E11.5 from the preoptic area (POA), after migration via the marginal zone, the subplate to the cortex and the hippocampus (Gelman et al. 2009; Gelman et al. 2011). Each population begins to migrate tangentially around E14 to E18, then switch to radial migration to invade the cortical plate between E18 and postnatal day 2 (P2) and finally reach the correct layer at P2 to P6.

Several waves of interneurons follow these steps, the first ones settling in deep layers of the cortex while the late ones invading the superficial layers (Bartolini et al. 2013). The existence and the relevance of these diverse paths are not clearly understood yet. Nonetheless, results obtained in a sub-class of SOM+ interneurons indicate that future interneurons could choose one road or another based on their mature morphology and final destinations (Lim et al. 2018b). On the other hand, neurons assigned to the hippocampus preferentially migrate tangentially through the marginal zone towards the *stratum lacunosum moleculare* and populate all layers of the hippocampus (Tricoire et al. 2011). Recent results suggest that the final concentration of intracellular PV decreases with each wave of interneurons, as early-born PV+ neurons display stronger PV signal than late-born PV+ neurons in the hippocampus, somatosensory cortex, and dorsal striatum (Donato et al. 2015).

After the disappearance of the MGE around E16, the ventral germinal zone of the lateral ventricle (VGZ) continues to generate interneurons, including the majority of ChC (even though a small fraction of them were generated earlier by the MGE) (Inan et al. 2012; Taniguchi et al. 2013). After the tangential migration around P0, neurons will cross the cortical plate around P2 to spread a little more at the cortical surface between P4 and P6 and finally invade cortical layers around P7 (Fig. 3) (Taniguchi et al. 2013). If some other subtypes of interneurons have postnatal sources to replenish their ranks (Inta et al. 2008; Wu et al. 2011; Riccio et al. 2012), it is yet to be proven for PV+ neurons. The migration of interneurons and their early diversification is a well-orchestrated mechanism that we are slowly beginning to grasp. As 2 recent reviews have dissected them in detail (Peyre et al. 2015; Lim et al. 2018a), we will not linger on those phenomena.

As reported by several authors, the first 2 postnatal weeks are characterized by a 40%–50% decrease in the density of different cell types, including interneurons (either in cortical or hippocampal structures) (López-Bendito et al. 2004; Tricoire et al. 2011). This phase corresponds to an increase in the whole brain volume, but stainings have proven that programmed cell death is also at play (Verney et al. 2000; Southwell et al. 2012; Denaxa et al. 2018; Priya et al. 2018). Indeed, the number of interneurons is to be tightly regulated: the loss of specific inhibitory cortical subpopulations is compensated by other subtypes or grafted interneurons to preserve the ratio of excitatory versus inhibitory neurons (Azim et al. 2009; Batista-Brito et al. 2009; Lodato et al. 2011; Denaxa et al. 2018). This reduction of inhibitory neuron density could be linked to network activity, as blocking NMDA receptors increases the number of apoptotic cells in the cortex (Roux et al. 2015). Also, brain regions showing higher network activities present reduced apoptosis of cortical neurons (Blanquie et al. 2017). A recent report shows that, in most interneurons, the network activity reduces postnatal cell death through activation of the Calcineurin protein (Priya et al. 2018).

#### Maturation

Regarding PV+ neurons, the first weeks of life are also marked by profound changes and maturations. Different studies have found that the fast profile of cortical and hippocampal BCs is reached between P7 and P25 (Itami et al. 2007; Doischer et al. 2008), after the switch from excitatory to inhibitory GABAergic signal (Rivera et al. 1999).

In the cortex, this modification of the firing profile is accompanied by changes in gene expression that could explain some electrophysiological parameters, e.g., 1) the down-regulation of *Kcnn2*, encoding for the small conductance Ca^2+^-activated K^+^ channel, which could favor the depressing profile observed from P10 or 2 the up-regulation of *Kcnc1* and *Kcnc2* genes (corresponding to the Kv3 potassium channel type) could account for the increased firing rates and reduced spikes from P10 (Okaty et al. 2009). Indeed, the blockade of Kv3 channels at P10 has little effect in PV+ neurons, whereas inducing a massive increase in IPSC in PCs at P18, confirming the upregulation of Kv3 channels and their effect on synaptic depression (Goldberg et al. 2011). Between P10 and P18, a K^+^ leak current appears, influencing the resting membrane potential (RMP) and the membrane resistance (R_m_) of PV+ neurons. At least K_ir_2 and K_2P_ channels participate, as RMP and R_m_ are modified upon application of specific blockers (Goldberg et al. 2011).

This critical period also corresponds to the settling of the Ca^2+^ managing system. Several Ca^2+^ channel subunits, which form low-voltage threshold (T-type: *Cacna1g*) or long-lasting activation (L-type: *Cacng4*, *Cacnb1*) channels, are down-regulated, presuming narrower regulation of Ca^2+^ flux. Indeed, the PV protein and the plasma membrane Ca^2+^-ATPase are also upregulated, inducing a tighter control of intracytoplasmic Ca^2+^ concentration (Okaty et al. 2009). The fastest calcium-sensor Syt2, used as BC marker in the visual cortex, is also upregulated from P10 to P18 (Sommeijer and Levelt 2012). Interestingly, electrical synapses between PV+ neurons can already be observed at P10, even if the arborization is not yet fully matured (Goldberg et al. 2011).

As for apoptosis, the correct maturation of PV+ interneuron also relies on network activity. In the cortex, GluN2C and D subunits of NMDA receptors help to establish the neuronal arborization (Hanson et al. 2019), whereas the GluN2A subunit is needed to face oxidative reactions and to establish the perineuronal net (PNN) (Cardis et al. 2018). The PNN is a specific type of extracellular matrix that surrounds several neurons and their dendrites. The PNN is composed of proteoglycan (PG), hyaluronan and smaller molecules synthesized by neurons and neighboring glial cells (John et al. 2006; Carulli et al. 2007). Different associations of PG can form these PNNs. PNNs in cortical BCs, (but not in ChC), are composed of chondroitin sulfate, keratan sulfate, and brevican PG (Wegner et al. 2003; Takeda-Uchimura et al. 2015; Favuzzi et al. 2017; Yamada and Jinno 2017). The PNN is settled from P10 to P30, mostly around cortical PV+ neurons and its establishment is influenced by received inputs and stimuli (McRae et al. 2007; Ye and Miao 2013; Ueno, Suemitsu, Murakami et al. 2017a). Some studies demonstrated that the magnitude, shape, and content of PNN associated with PV+ neurons can vary between the regions of the brain (Yamada and Jinno 2013; Ueno, Suemitsu, Okamoto et al. 2017b). PNNs are well known to influence synaptic plasticity and may have a subtler role than only a physical barrier preventing synapse establishment. PNNs can bind molecules such as β-Integrin in the hippocampus or Sema3A in the cortex, respectively, preventing their promoting role in spine formation (Orlando et al. 2012) or enabling their inhibitory action (Vo et al. 2013). Indeed, the PNN may impact the cortical neuron without complete net digestion, but rather via the change of sulfation pattern involved in protein binding (Miyata et al. 2012; Miyata and Kitagawa 2016). PNNs also modulate receptor activity by acting 1) directly as a physical fence, as demonstrated for the AMPAR GluA1 and 2 receptors (Frischknecht et al. 2009), or 2) indirectly by sequestrating or accumulating partners. For example, the neuronal-pentraxin 2 (NP2 encoded by the *Narp* gene) modulates GluA4 in hippocampal PV^+^ neurons after being secreted, only in the presence of PNN (Chang et al. 2011). Finally, PNNs are also involved in the trafficking and the clustering of K^+^ channel Kv1.1 and 3.1 in the hippocampus (Favuzzi et al. 2017).

Concerning maturation, less information is known about ChCs. Some papers have highlighted a slower establishment of synapses and FS properties in mouse cortical ChCs, compare to BCs (Miyamae et al. 2017; Pan-Vazquez et al. 2020). Nonetheless, one specific feature of maturating ChCs is still under debate: ChCs may remain excitatory longer than other inhibitory neurons. At first, the high intraneuronal Cl^−^ concentration makes GABA inputs depolarizing, and consequently all “inhibitory” neurons remain excitatory. Around P7 in mouse, the expression of symporter KCC2 rises and shifts the Cl^−^ gradient to induce the hyperpolarizing effect of inhibitory neurons (Rivera et al. 1999; Ben-ari 2002). Yet, several teams have observed depolarizing input coming from ChCs in cortices and hippocampi of rodent between P15 and P35 (Szabadics et al. 2006; Khirug et al. 2008; Woodruff et al. 2011). As interneurons connect to different parts of their targets, those observations could be explained by local change of Cl^−^ concentration gradient. In mouse cortical neurons, KCC2 expression rises later in the AIS, which could explain why ChCs would remain depolarizing longer than BCs (Rinetti-Vargas et al. 2017; Pan-Vazquez et al. 2020).

The late maturation of PV^+^ neurons, simultaneous to the establishment of the synaptic network, makes them a pivotal actor in neurodevelopmental and neurodegeneration diseases (van Bokhoven et al. 2018; Ferguson and Gao 2018; Wen et al. 2018).

### Parvalbumin Neurons in Epilepsy

PV+ neurons display a strong ability to decrease the global brain excitability and are thought to play a role in epilepsy. Epilepsy is a group of disorders commonly characterized by the recurrent appearance of seizures, which consist of abnormal and highly synchronous brain activity and AP discharges. The cause of these diseases can sometimes be identified (i.e., genetic, traumatic, hemorrhagic …), but is unknown in most cases (i.e., idiopathic or cryptogenic epilepsy). Epilepsy is a chronic disease with very acute expression, and patients suffering from epilepsy are treated to control seizures onset rather than handling the cause of the disease. Epilepsy being an evolving disorder (frequency and severity of seizures can change over time), treatments have to be regularly adjusted. Moreover, one-third of epileptic cases are “refractory” or “intractable” to current antiepileptic treatments (Chen et al. 2018; Faraji and Richardson 2018).

**Figure 4:**
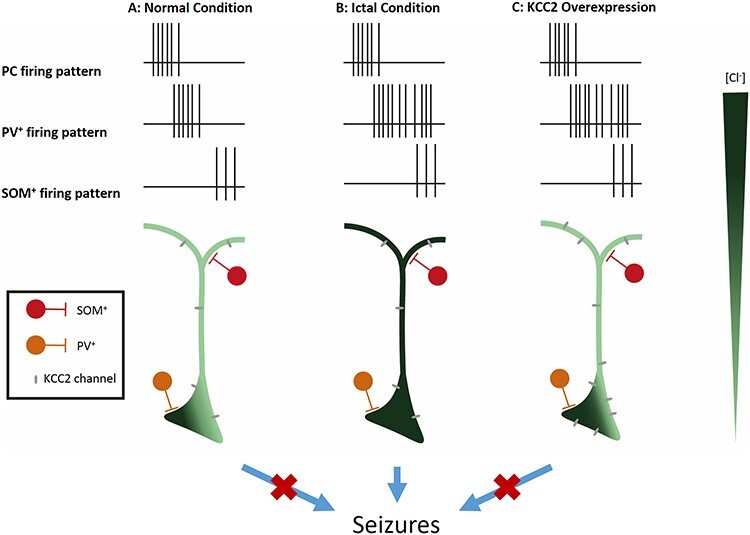
Hypothesis of network mechanism potentially giving rise to seizure. The pyramidal neuron (in green/black) receives input from Som + neurons (in red) and PV neurons (in orange). Vertical bars represent activities of the corresponding neuron along the horizontal timeline. The green gradient represents Cl^−^ ion concentration inside the pyramidal neuron.

Epilepsy is thought to be linked to a failure of the excitatory to inhibitory balance (E/I balance), and PV+ neurons were rapidly assumed to participate in ictogenesis (seizure onset) or epileptogenesis (epilepsy appearance and evolution). Indeed, several studies have shown that PV+ neuron density is reduced in epileptic tissue of animal models, as well as in human patients (Zamecnik et al. 2006; Kuruba et al. 2011; Marx et al. 2013; Nakagawa et al. 2017; Cameron et al. 2019; Alhourani et al. 2020). Interestingly, a recent paper observed an increased mitochondrial fragmentation in rat PV+ neurons following induced *status epilepticus*. Moreover, they show that reducing mitochondrial fission with chemical inhibitor mitigate PV+ neuron loss (Kim and Kang 2017). However, other teams observed a normal number of PV+ neurons with an altered electrophysiological profile or morphology in different animal models with induced seizures (Sun et al. 2007; Gu et al. 2017; Miri et al. 2018). Following experimental observation in mice, it has also been proposed that the parvalbumin protein itself could be lost, rather than the whole PV+ neuronal population, due to Ca^2+^ overload and excessive recruitment of interneuron (Wittner et al. 2001; Wittner and Maglóczky 2017). Given the diversity of causes and symptoms of epilepsy, as well as animal models, finding a consensus is a struggle. Anyway, these observations suggest an increased vulnerability of PV+ neurons in epilepsy but give little information on the role of PV+ neurons in epileptogenesis.

Many genetic mutations in PV+ neurons have been proposed to play a role in epilepsy and were even detected in patients. Many of those have been reviewed by Jiang, Lachance and Rossignol in 2016 (Jiang et al. 2016). In this paper, authors analyzed alterations of PV+ neurons in terms of migration, maturation, excitability, or connectivity. But, from 2016, the list is still growing and we will thus focus here on several recent findings. Ankyrin-G participates in the clustering of ions channels in nodes of Ranvier and the AIS. In absence of the PV+ specific ankyrin-G isoform 1b, PV+ neurons have reduced excitability, leading to seizures and behavioral alteration associated with bipolar disorder (Lopez et al. 2017). CNTNAP2, a protein belonging to the pre-synaptic cell-adhesion protein family neurexin, is associated with many neurological disorders, including epilepsy. A team recently showed that FS cortical neurons mutated for CNTNAP2 present altered AP width or intern spike interval when transplanted into wild-type mice cortices (Vogt et al. 2018). Even more recently, it was shown that the absence of NHE1 (the Na^+^/H^+^ exchanger expressed in neurons and astrocytes) in mouse hippocampal PV+ neurons decreases the frequency and increases the amplitude of mIPSC, probably by affecting the loading of GABA vesicles (Bocker et al. 2019). On the other hand, Soh and colleagues associated an increase of spontaneous IPSC frequency with a shorter latency of chemically induced seizures in mice mutated for the K^+^ channel KNCQ2 (Soh et al. 2018), implying that stronger inhibition could also lead to seizure susceptibility.

Despite the classical consideration of epilepsy, being either due to increased excitability of glutamatergic cells or reduced inhibition and unleashing of excitatory cells, increasing data highlights the role of increased activity of inhibitory neurons in seizures. Indeed, several articles have described a modified activity of PV+ neurons followed by a “depolarization block” event right before the seizure onset in rodents (Fujiwara-Tsukamoto et al. 2004; Ziburkus et al. 2006; Grasse et al. 2013; Parrish et al. 2019). These depolarization block (DB) events correspond to a high-frequency train of truncated AP. Other teams have reported an increase of GABA release by hippocampal PV+ neurons during high-frequency train in kindled mice model (Hansen et al. 2018), or an increased firing frequency of hippocampal PV+ neurons during pre-ictal phase in chemo-induced seizures mice model (Miri et al. 2018). Interestingly, a reduction of VIP+/CR+ contacts made on OLMs, BiCs, and BCs was reported in the CA1 in pilocarpin mice model. While all interneurons displayed a reduction of spontaneous IPSC frequency, only IPSC amplitudes recorded in BiCs or BCs were decreased after light-evoked VIP+ neuron stimulation. This particular impact on BCs could explain the specific increased activity of PV+ reported by Hansen and Miri (David and Topolnik 2017). The intensity of these inhibitory currents could trigger a massive accumulation of Cl^−^ inside post-synaptic neurons and an increase of extracellular K^+^ concentration, probably due to K^+^/Cl^−^ symporter (Fujiwara-Tsukamoto et al. 2007). Once high, the extracellular K^+^ concentration eases the depolarization of the excitatory cell, completely free to fire as inhibitory cells are not active anymore because recovering from the DB. Furthermore, it has been demonstrated that PV+ neurons are recruited before SOM+ neurons and that these 2 populations could be needed to dam seizures (Parrish et al. 2019).

When excessive excitation stimulates interneurons (Fig. 4), the first-line cells are PV+ neurons targeting the soma of the glutamatergic neurons. PV+ neurons strongly and quickly induce inhibition, leading to a surge of Cl^−^ inside the somatic compartment of the PC, which could sustain ion entry for a while, thanks to K^+^/Cl^−^ symporter like KCC2. Once PV+ neurons are depressed, the second line is represented by SOM+ neurons, which then prevent massive excitation of glutamatergic neurons. SOM+ neurons provide a long-lasting inhibition, maintaining the non-responsive state of PCs that PV+ neurons have begun to build. If PV+ neurons remain active or reactivate, they could saturate PC in Cl^−^ that may start diffusing, overtaking KCC2 capacity. This would impact the dendritic domain, inducing an increase of extracellular K^+^ where PC receives excitatory drive. SOM+ neurons will not be sufficient to maintain inhibition, and the PC will begin to frenetically fire. Another possibility could involve extrasynaptic GABA receptor, as recently found in SOM+ neurons (Bryson et al. 2020). This could explain why PV+ neurons have been reported as a “double agent” in ictogenesis (Shiri et al. 2016; Wang et al. 2017; Lévesque et al. 2019). It has been reported that overexpression of KCC2 prevents the pro-ictal effect of PV+ neurons when stimulated 2 s after a seizure (Magloire et al. 2018). The increased number of KCC2 channels on PC could prevent the spreading of Cl^−^ and keep the increase of extracellular K^+^ minimal around dendrites.

## Conclusion

The last decades have brought to light the crucial role that interneurons play in neurotransmission. Although a minor population, these inhibitory neurons are key fine tuners that regulate signals of projection neurons and have been closely examined after years of being wrongly omitted. As our knowledge piles up, we unravel the broad diversity of interneurons and gain new insights into their various structural, biochemical, and functional aspects. Parvalbumin-positive (PV+) interneurons were characterized based on their specific fast-spiking profile, but beyond this specific characteristic, we keep collecting evidence that indicates they are probably much more diverse than we think. The variety of their neurite arborization, ion balance, synaptic components, and spatial distribution probably confers them miscellaneous functions that we slowly start to grasp.

Epilepsy physiopathology is one example of this specific functionality of these PV+ neurons and by itself, it is not an exception to this rule of diversity. Indeed, it includes a large phenotypical spectrum and is associated with a number of dysregulated molecular and biochemical mechanisms. Getting back to PV+ neurons, growing evidence from the literature sheds light on their role(s) on neuronal networks as they are particularly wired and equipped for influencing those grids. Moreover, these neurons could be the ideal targets for treatment: only a tiny amount of drug acting on this small cell population would be able to trigger a massive effect. Still, this powerful PV+ neuron machinery could turn out to be both ally and enemy for a correct brain function, and the genuine role of these neurons in ictogenesis is yet unclear. Whether an inadequate firing profile is directly linked to seizure, or whether their firing profile is itself impacted by dysregulated essential cellular processes (e.g., energy, transport, cytoskeletal remodeling, etc.) indirectly causing seizure, are questions that should be further investigated.

Much remains to untangle in order to fathom the way PV+ interneurons play in harmony with neighboring neurons, to identify which player in out of tune in pathological conditions, and to ultimately succeed in conducting this orchestra to play a symphony without a false note.

## Notes

We thank Estelle Willems for critical reading and orthographic revision of this manuscript. *Conflict of Interest*: None declared.

## Funding

The Fonds de la recherche scientifique (FRS-FNRS), the Fond Léon Fredericq, and the University of Liège.
